# Rapid Determination of Fish Species of Raw and Heat-Treated Fish Meat Using Proteomic Species-Specific Markers

**DOI:** 10.17113/ftb.63.03.25.8512

**Published:** 2025-08-31

**Authors:** Alena Meledina, David Straka, Filip Soucek, Tatiana Anatolievna Smirnova, Stepanka Kuckova

**Affiliations:** 1 Department of Biochemistry and Microbiology, University of Chemistry and Technology, Prague, Technicka 3, 166 28 Prague 6, Czech Republic; 2 Department of Chemistry and Chemistry Education, Charles University, Prague, M.D. Rettigove 4, 110 00 Prague 1, Czech Republic

**Keywords:** fish meat, fish species, species-specific *m/z* values, peptide markers, mass spectrometry, food authentication

## Abstract

**Research background:**

The main problem regarding the authenticity of fish meat lies mainly in misleading labelling or substitution of species, like the replacement of valuable fish meat with species of lower value or species originating from illegal fishing. For these reasons, the need has arisen for adequate analytical methods to detect food fraud.

**Experimental approach:**

The aim of this study is to differentiate six fish species—carp, mackerel, pike, pollock, salmon and trout—based on differences in their protein composition using two mass spectrometry methods. Matrix-assisted laser desorption/ionization–time ff flight mass spectrometry (MALDI-TOF MS) was employed to identify characteristic species-specific *m/z* values to differentiate raw and cooked fish meat. Additionally, liquid chromatography–electrospray ionization–quadrupole–time tf flight (LC-ESI-Q-TOF) was used to determine specific amino acid sequences in carp and salmon, selected as model species.

**Results and conclusions:**

Distinct species-specific *m/z* markers were identified for all six fish species, enabling their differentiation in both raw and processed form. In carp and salmon, hundreds of peptide sequences were detected, leading to the identification of a panel of peptide markers that determine both the fish species and the type of meat processing. The results confirm that mass spectrometry-based proteomic approaches can serve as effective tools for the authentication of fish meat.

**Novelty and scientific contribution:**

This study shows that it is possible to use two complementary mass spectrometry techniques for reliable and rapid authentication of fish species. The identification of specific peptide markers and species-specific *m/z* values contributes to the improvement of food authenticity control and provides a powerful approach to the detection of fish meat adulteration.

## INTRODUCTION

The main problem regarding the fish and seafood authenticity seems to be misleading labelling or species substitution (replacing a more expensive fish with a cheaper one). The labelling of the fish species is a mandatory requirement in the vast majority of legislative regulations. Especially in processed products, where visual identification is not possible in some cases, the identity of the animal can be falsified. There is ususally an economic incentive to replace valuable material with species of lower value or species originating from illegal fishing. Another problem is the fact that many species of seafood are sold under a collective name (*1*–*4*).

The methods used to authenticate meat are generally based on DNA or protein analysis. Molecular techniques based on DNA analysis have undergone enormous development in recent decades. They overcome some limitations of methods based on protein analysis, such as protein denaturation during heat treatment of meat, which can lead to changes in the antigenicity of molecules and their electrophoretic mobility (*5*, *6*). However, similar challenges can occur with DNA barcoding when distinguishing closely related species. For example, although DNA barcoding of mitochondrial cytochrome c oxidase subunit 1 (COI) successfully identified 14 of 16 freshwater fish species from Lake Wivenhoe (Queensland, Australia), two undifferentiated species from the family Terapontidae, which have identical COI gene sequences, could not be distinguished using this method (*7*). This highlights a limitation of DNA barcoding in distinguishing closely related species, a problem that may also exist in protein-based methods. Digital polymerase chain reaction (dPCR) and its modified form droplet digital PCR (ddPCR) are the oldest used DNA amplification techniques that use a water-oil emulsion drop system (*5*, *8*). Doi *et al.* (*9*) used ddPCR for the detection of environmental DNA (eDNA) originating from an invasive fish species – the bluegill sunfish (*Lepomis macrochirus*). Furthermore, ddPCR has been used to identify and quantify the highly valued silver pomfret (*Pampus argenteus*), whose adulteration is a serious problem worldwide (*10*), and also to analyse marine products from cod (*Gadus chalcogrammus*), which is of great commercial importance (*11*). PCR analysis with restriction fragment length polymorphism (PCR-RFLP) is the most widely used method for the identification of meat species including fish (*12*–*14*). This assay has been optimised to distinguish three closely related gadoid fish species: Alaska pollock, Pacific cod and Atlantic cod in commercial seafood products (*15*). Lin and Hwang (*16*) successfully identified eight tuna species in canned products using this technique. Species-specific PCR can be used to identify the taxonomic origin of fish meat and seafood products. For example, Kim *et al.* (*17*) differentiated three related grouper species: *Epinephelus septemfasciatus*, *E. bruneus* and *E. akaara*. Multiplex PCR is a method that enables simultaneous identification of several species. This technique has been used to distinguish seven Clupeiform species, including several economically important fish such as herring and sardines (*18*). Real-time PCR has also been used in the authentication of fish meat. The method was developed for the differentiation and quantification of two closely related tuna species (bigeye tuna – *Thunnus obesus* and yellowfin tuna – *Thunnus albacares*) in canned products (*19*). The combination of real-time PCR and multiplex PCR was used for the identification of eight ecologically and economically important freshwater fish species (*20*).

Traditional protein analysis techniques include immunological, chromatographic, spectroscopic and electrophoretic methods. The U.S. Food and Drug Administration (FDA) maintains the Regulatory fish encyclopaedia (*21*), which serves as a repository of data on protein analyses for fish identification. Traditional protein methods face challenges due to the denaturation or degradation of proteins that often occurs during sample preparation. This makes these methods generally unsuitable for the identification of proteins in processed meat. However, in some studies, fish species were identified with the enzyme-linked immune sorbent assay (ELISA) using antibodies against muscle proteins. It involved distinguishing canned sardines from other fish such as herring, mackerel, anchovy (*22*) and identifying individual species of flatfish (*23*). Red snapper was also identified using this technique (*24*), and raw and processed grouper meat was distinguished from cheaper fish species (*25*).

Sodium dodecyl sulphate–polyacrylamide gel electrophoresis (SDS-PAGE) was used by a group of Pineiro *et al.* (*26*) to differentiate of 15 species of raw and cooked fish. Also, the combination of SDS-PAGE with isoelectric focusing was successful in the identification of species of unknown samples (*27*, *28*). Martinez and Jakobsen (*29*) used two-dimensional electrophoresis (2-DE) to investigate the authenticity of fish and shrimp and also to assess their freshness using separated myofibrillar proteins. Berrini *et al.* (*30*) distinguished four species of fish, which were sold under the same trade name "perch", using a method focusing on sarcoplasmic proteins.

High-performance liquid chromatography (HPLC) methods for determining the type of meat typically rely on analysing protein, peptide or amino acid profiles unique to different types of meat. For instance, 31 fish species were distinguished using HPLC analysis of water-soluble sarcoplasmic muscle proteins (*31*). However, it was found that heat treatment (cooking) had a relatively significant effect on the quality of the chromatograms (*31*). Chou *et al.* (*32*) developed a method applicable to fresh and cooked meat for the routine discrimination between meat products from 15 common animal species (mammals, birds and fish) based on HPLC with electrochemical detection using copper nanoparticles.

Currently, mass spectrometry (MS) techniques play a key role in the analysis of proteins and peptides in food, including the investigation of the authenticity of meat and meat products. For example, Volta *et al.* (*33*) distinguished three freshwater fish meat species (*Alosa agone*, *Coregonus macrophthalmus* and *Rutilus rutilus*) based on the differences in the spectra of muscle tissue using MALDI-TOF MS.

In this work, the proteomic approach is tested using MALDI-TOF and LC-ESI-Q-TOF mass spectrometry to distinguish six selected fish species (three marine and three freshwater). Samples were digested with trypsin without additional protein extraction before proteomic analysis using both mass spectrometry methods. This approach was successfully used for the preparation of different samples of taxonomical origin (*34*, *35*). The obtained data were evaluated using the PostgreSQL database system created in our laboratory, which was accessed using the pgAdmin interface (*36*). Species-specific markers (*m/z* values and amino acid sequences) enabling reliable identification of fish were found. The possibility of distinguishing between raw and heat-treated meat is also investigated. The aim is to distinguish between individual species even in the case of heat treatment, and to try to distinguish between raw and heat-treated meat of the same species.

## MATERIALS AND METHODS

### Reagents and materials

Acetonitrile (ACN) of LC-MS grade, 2,5-dihydroxybenzoic acid (DHB), formic acid (FA) and trifluoroacetic acid (TFA, suitable for HPLC-MS) were purchased from Sigma-Aldrich, Merck (Burlington, MA, USA). Ammonium hydrogen carbonate (AHC, suitable for HPLC-MS) was obtained from Lachema (Brno, Czech Republic). Peptide calibration standard II was purchased from Bruker Daltonics (Bremen, Germany). Pierce trypsin protease MS grade was obtained from Thermo Fisher Scientific (Waltham, MA, USA). The commercially available reverse phase ZipTip C_18_ pipette tips were purchased from Millipore Corporation (Burlington, MA, USA). The water was purified with a Milli-Q water purification system from Millipore Corporation.

### Reference samples of fish meat

The meat from six selected fish species was analysed. Among them were three freshwater species: common carp (*Cyprinus carpio*), northern pike (*Esox lucius*), rainbow trout (*Oncorhynchus mykiss*) and three marine species: Atlantic mackerel (*Scomber scombrus*), Alaska pollock (*Theragra chalcogramma*) and Atlantic salmon (*Salmo salar*). Freshwater fish species were purchased from the local fish store named Štičí líheň ESOX spol. s. r. o. in Tábor (Czech Republic), marine species were from wholesale chains Albert and Lidl. Three individuals of each species (biological replicates) were analysed in this work.

Two types of samples were prepared for each fish: raw and cooked meat. The heat-treated samples were prepared by boiling 1–2 g of cut fish meat in boiling water for 10 min. Both types of samples were then stored in a freezer (-80 °C). These primary samples were later used for the analysis. Three samples (technical replicates) were weighed (approx. 1 mg) for each type of fish. Nine samples of raw meat and nine samples of cooked meat from each fish species were prepared for the analyses.

### Sample preparation

A mass of 1 mg of each sample was digested in 20 µL of 50 mM AHC containing 0.02 mg/mL of trypsin at 37 °C with constant shaking for two hours. After two hours, the cleavage was terminated by adding 1 μL of 10 % TFA solution to a final amount of 0.5 % TFA. After the trypsin digestion, the samples were purified and concentrated on reverse phase ZipTip C_18_. After purification, 10 μL of each purified sample were obtained.

### MALDI-TOF MS measurements and data acquisition

A volume of 2 µL of purified peptide sample was mixed with 7.5 µL of DHB matrix solution (8.5 mg of DHB in 0.5 mL of a mixed solvent: water, acetonitrile and 0.1 % TFA). A volume of 1.3 μL of the resulting mixture was spotted thrice on the stainless steel MALDI target and air-dried. Mass spectra were acquired using MALDI-TOF Autoflex Speed mass spectrometer (Bruker Daltonics) equipped with an Nd:YAG laser (355 nm) in positive reflector mode. The obtained spectra contained peaks in the 900–4500 *m/z* interval. The corresponding spectrum was obtained from a total of 7000 shots for each spot.

### LC-ESI-Q-TOF MS conditions and data acquisition

Measurements were carried out using UHPLC Dionex Ultimate3000 RSLC nano (Dionex, Bremen, Germany) connected to ESI-Q-TOF Maxis Impact mass spectrometer (Bruker Daltonics). Purified and air-dried samples (after trypsin digestion, see Sample preparation) were dissolved in 10 μL of a mixture of 3 % acetonitrile and 0.1 % formic acid. A volume of 3 µL of the solution was loaded into an Acclaim PepMap 100 C18 trap column (100 µm×2 cm, size of reverse phase particles 5 µm; Dionex) and 3 % of mobile phase B with a flow rate of 5 µL/min was run for 7 min. The peptides were then eluted from the trap column into Acclaim PepMap RSLC C18 analytical column (75 µm×150 mm, size of reverse phase particles 2 µm; Dionex) using the following gradient: 0–5 min 3 % B, 5–35 min 3–35 % B, 37 min 90 % B, 37–50 min 90 % B, 51 min 3 % B, 51–60 min 3 % B. The mobile phase A consisted of 0.1 % formic acid in water and mobile phase B of 0.1 % formic acid in acetonitrile. The flow rate during gradient separation was set at 0.3 µL/min. The peptides were eluted directly to an ESI source – Captive spray (Bruker Daltonics). Measurements were carried out in positive ion mode with a precursor selection in the range of 400–1400 Da; up to 10 precursors were selected for fragmentation from each MS spectrum.

MS spectrum was recorded every 3 s, MS/MS spectra were collected at 4–16 Hz depending on precursor intensity. Dynamic precursor exclusion was set to 1 min, preferred number of precursor charges was 2–5. Singly charged precursors were excluded from fragmentation. Collision-induced MS/MS spectra were recorded in the range of 50–2200 *m/z*. Mass spectra were extracted by DataAnalysis 4.1 (Bruker Daltonics) and loaded into Proteinscape 4.2 (Bruker Daltonics) and later into Mascot 2.4.1 (Matrix Science, Boston, MA, USA), which was used for protein identification. The identification was carried out against a single-species database containing the proteome of the investigated species (*Cyprinus carpio* and *Salmo salar* were downloaded from the UniProt website on 4 April 2022) (*37*), which was supplemented with common laboratory contaminants. The following identification parameters were used: enzyme trypsin (one missed cleavage site was allowed), oxidation of methionines as a variable modification, accuracy of assigning precursors of 10 ppm and fragments of 0.05 Da. Identified peptides and proteins were filtered to maintain a false positive identification rate of 1 %. All samples were analysed by LC-ESI-Q-TOF MS in three repetitions to obtain characteristic peptide profiles.

### Searching for species-specific markers

To distinguish individual types of fish meat, species-specific markers were identified as *m/z* or peptides that occurred with certain frequency in the spectra obtained from one species but were absent in the spectra of other fish species. The frequency represents a number of spectra in which the particular *m/z* values or amino acid sequences occurred. Then these peptides or *m/z* values can be considered species-specific markers within the selected group of fish. Mass spectra were processed using two complementary methods: MALDI-TOF and LC-ESI-Q-TOF.

For MALDI-TOF data processing, the mMass software, v. 5.5.0 was used (*38*). The involved spectrum smoothing, baseline correction and manual peak selection, where 80–110 peaks were selected for each MALDI-TOF spectrum. The *m/z* values from the spectra obtained for a single species were then recorded in Microsoft® Excel® for further analysis.

To manage and analyse the extensive data, we used the PostgreSQL object-relational database system (v. 2022.4.4) with pgAdmin 4 (v. 6.21), an open-source graphical administration tool for PostgreSQL (*36*). The analysis focused on identifying *m/z* values that appeared consistently across spectra for each species. This process was adjusted to include only peaks present in a specific frequency, set in this study to a minimum of 23 out of 27 MALDI spectra (three individuals, three technical replicates and three spots per technical repetition).

Similarly, Excel data files containing the results (identified peptides and their corresponding proteins) from LC-ESI-Q-TOF MS were processed using comparable steps. Species-specific peptides were identified as those consistently present in all nine Excel data files corresponding to a single species (three individuals and three technical replicates, where each technical replicate was injected once).

## RESULTS AND DISCUSSION

### Results of MALDI-TOF mass spectrometry

MALDI-TOF MS measurements were conducted on all six fish species, both raw and cooked samples. This analysis identified *m/z* values that serve as species-specific markers and characterise each fish species.

### Searching for species-specific markers by MALDI-TOF MS

**Fig. 1:**
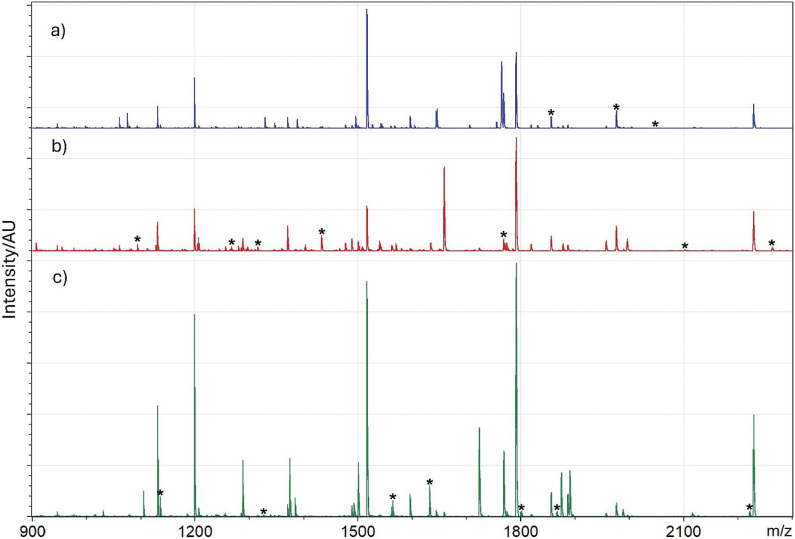
MALDI-TOF MS spectra of: a) salmon (blue), b) carp (red) and c) pollock (green). Characteristic *m/z* values for raw meat are indicated with asterisk (*)

The list of peptides (*m/z* values) was obtained for each fish species using the data evaluation described in *Searching for species-specific markers*. The *m/z* values were determined separately for raw samples across all species and in a separate analysis for cooked samples. In the next step, values were compared between individual species to find characteristic values for one specific species, *i.e.* a given *m/z* value that occurred in one species, but not in any other. Table 1 lists the characteristic values for raw and cooked meat samples. Fig. 1 shows the MALDI-TOF MS spectra for carp, pollock and salmon, highlighting the differences in their characteristic *m/z* values. These spectra serve as a visual representation of some species-specific markers identified in our analysis.

**Table 1:** Characteristic *m/z* values for raw and cooked meat of individual types of fish (underlined values are the same for raw and cooked fish meat). The m/z values were determined independently for raw samples across all species and in a separate analysis for cooked samples

**Type of meat**	**Fish species**	***m/z***								
**Raw**	Carp	1093.7	1263.6	1309.7	1432.7	1770.8	2101.1	2263.3		
	Mackerel	934.6	1269.7	1380.8	1397.9	1411.8	1463.7	1777.0	1836.0	1932.2
		2023.2	2095.2	2291.4	2638.6	4048.3				
	Pike	1226.6	2587.5	4165.5						
	Pollock	1142.8	1325.8	1564.3	1632.2	1800.5	1866.6	2221.6	2553.6	2635.2
	Salmon	1868.2	1980.4	2059.1						
	Trout	1109.9	3175.6							
**Cooked**	Carp	1093.7	1137.5	1180.5	1633.6	1770.8	2101.1	2185.0	2317.1	2484.2
		2732.1								
	Mackerel	934.6	1050.6	1115.7	1239.8	1269.8	1296.8	1308.7	1380.9	1397.9
		1411.8	1506.8	1725.2	1777.1	1838.2	1932.1	2291.5	2392.4	
	Pike	1355.9	2587.5	2723.7	3458.3	4165.5				
	Pollock	1142.9	1325.9	1339.0	1384.0	1409.1	1561.2	1632.2	1774.6	1780.7
		1800.5	1866.7	1890.6	1895.7	2221.7	3183.5			
	Salmon	2059.1	3291.4							
	Trout	1344.6	1565.7	3175.6						

 The differentiation of fish species by MALDI-TOF MS using species-specific protein patterns has been shown previously (*39*–*42*). These studies used a simpler approach without trypsin digestion and analysed extracted proteins directly. Enzymatic cleavage was only used in a subsequent step for the identification of protein biomarker. This method has proven effective for distinguishing species, even without the need for tryptic digestion, particularly for species that are not closely related, such as the six species in our study.

 Our approach differs fundamentally in the use of trypsin digestion prior to MALDI-TOF MS measurements. This technique, known as in-sample digestion, fragments proteins into peptides, resulting in spectra with a broader range of information and a higher resolution. While the approach of Mazzeo *et al.* (*39*) identified markers in the >11 000 *m/z* range, corresponding to small proteins like parvalbumins, or Stahl and Schroeder (*40*), who collected spectra in the mass range 2–20 kDa, our analysis focuses on the 900–4500 Da range to capture differences in the whole proteome rather than targeting specific proteins.

 Spielmann *et al.* (*42*) explored the use of MALDI-TOF MS for processed meat and developed a database of species-specific fish proteins using the Biotyper tool. While effective, the Biotyper tool is proprietary and requires a paid license, making it less accessible. In contrast, the approach described in this paper utilises PostgreSQL, an open-source database system, which not only reduces costs but also provides broader accessibility to researchers and laboratories.

 Although the six tested species could probably be differentiated using simpler methods, this method shows strong potential in the context of heat-treated fish meat, where the higher resolution and broader marker identification provide added value. By focusing on peptide-level markers and leveraging accessible tools, the shown approach offers a cost-effective and innovative alternative for fish meat authentication.

 Markers for different types of cooked fish meat were then searched in the same way. In this analysis, at least two specific markers were found for each species. The list of characteristic values of the cooked meat samples is shown in Table 1. A slightly bigger number of characteristic values was found for cooked meat. This can be explained by the fact that proteins in their native structure are not as easily accessible to trypsin cleavage in some positions as the loosened, thermally denatured proteins.

 Some *m/z* values are identical for raw and cooked fish meat. These values can be considered as characteristic markers for the respective species regardless of the type of meat processing (underlined values in Table 1). The other half of the *m/z* values, which are not the same for raw and cooked meat, proves that some markers differ depending on the type of processing to distinguish the respective species. The obtained results prove the feasibility of the method also for cooked meat and at the same time confirm the stability of some MALDI-TOF MS markers during heat treatment (markers found simultaneously for raw and cooked meat), as mentioned in the literature (*39*, *40*).

The next step was to search for markers that could distinguish between raw and heat-treated meat of the same species. In this phase, we therefore focused on the *m*/*z* values that differentiate the raw and cooked form of a particular fish—for example, markers unique to raw carp *versus* cooked carp, and by analogy the other species analysed. The search for markers was carried out sequentially for all analysed species and the results are shown in Table 2. The results show that the raw fish meat samples contained more specific markers than their cooked counterparts. The underlined values represent peaks that are already included in Table 1 and serve as markers for distinguishing between the raw or cooked meat species. These markers have a high discriminatory value and when present in a sample, can determine not only the type of fish meat but also its type (raw or heat treated). Such markers have been found for raw meat of carp, mackerel and pike, in the case of cooked meat of carp and mackerel.

**Table 2:** Characteristic *m/z* values for differentiating raw and cooked fish meat of individual fish species (underlined values are usable to characterise the type of fish as well as the type of processing). Only those *m*/*z* markers that distinguish the raw and cooked form of the same species—*e.g.* raw carp *versus* cooked carp—were considered in this comparison

**Fish species**	**Type of meat**	***m/z***						
**Carp**	raw	1050.6	1127.7	1309.8	1383.7	1500.8	1561.9	1569.9
		1877.3	2480.2					
	cooked	1661.6	2184.9	2446.1	2732.1			
**Mackerel**	raw	914.5	1028.6	1127.7	1643.9	1650.9	2023.2	2115.1
	cooked	909.6	1239.8	2216.4				
**Pike**	raw	1127.7	1226.8	1358.9	1402.8			
	cooked	1279.9	1908.4					
**Pollock**	raw	1254.9	1740.0					
	cooked	1470.0						
**Salmon**	raw	1002.5	1050.6	1067.6	1127.7	1269.7	1296.8	1339.7
		1397.8	1411.8	1506.9	1682.0	1777.0	1932.0	4048.3
	cooked	1135.5	1237.6	1476.7	1488.7	1754.8	1854.7	2003.0
		2384.2	2512.4					
**Trout**	raw	1240.8	1358.8	1536.1	1560.0	1705.0		
	cooked	1400.6	2118.9	2406.1	2438.0	3414.6	3471.7	

### Results from LC-ESI-Q-TOF mass spectrometry

The LC-ESI-Q-TOF analyses were performed only on carp and salmon samples, both raw and cooked (a total of 36 samples were analysed – nine for each type of processing of these species). The reason for this was that in the used UniProt protein database (in which the peptides and proteins are searched) the fish species category is not complete. Therefore, only the two species whose protein sequences are available in the database were selected. This method can detect the amino acid sequences of peptide fragments and identify the protein from which the fragments originate. This provides more accurate data than the *m/z* values usually obtained by MALDI-TOF MS.

#### Identified proteins

Over 100 proteins (100–150) detected based on at least two peptides (with a length from 7 to 30 amino acids) were identified in each sample. The same proteins were mostly identified in cooked and raw meat of the same species, but the number of peptides found differed between them, which again indicates slightly different cleavage of the heat-treated proteins by trypsin. Table 3 shows selected identified proteins in raw and cooked carp meat. Similarly, Table 4 shows the proteins found in salmon.

**Table 3:** Selected proteins identified in raw and cooked carp meat

**Access code**	**Protein**	***N*(peptide)**	
raw	cooked
**A0A8C1U661**	Myosin heavy chain, fast skeletal muscle-like	124±9	122±9
**A0A8C2BNT8**	Myosin, heavy chain b	65±4	60±6
**A0A8C1U0K1**	Nebulin	59±9	86±17
**A0A8C1WNY8**	ATPase sarcoplasmic/endoplasmic reticulum Ca^2+^ transporting 1, like	35±2	38±5
**A0A8C1N3F8**	Creatine kinase M-type	35±3	37±3
**A0A8C1T5E4**	Actin alpha 1, skeletal muscle	33±4	36±4
**A0A8C1X499**	Actinin alpha 3b	31±3	32±3
**A0A8C1USJ2**	Phosphorylase, glycogen, muscle A	29±4	29±5
**A0A8C1V0Y0**	Myosin regulatory light chain 2, skeletal muscle isoform-like	25±2	-
**A0A2U9IYA4**	Fructose-bisphosphate aldolase	23±3	23±4
**A0A8C1J152**	Myosin light chain 3, skeletal muscle isoform-like	22±3	16±2
**A0A8C1FTE8**	Alpha-tropomyosin	22±1	19±3
**A0A8C1W232**	Myosin light chain 1, skeletal muscle isoform-like	17±2	-
**A0A8C1TSP1**	EF-hand calcium binding domain 7	16±3	-
**A0A8C1S5P5**	Calsequestrin 1a	8±1	-
**A0A8C1RRE4**	Glyceraldehyde-3-phosphate dehydrogenase	-	24±2
**A0A8C1S397**	Enolase 3 (beta, muscle)	-	19±5
**A0A8C1ZWS8**	Myosin binding protein C, fast type b	-	19±2
**Q8UUS2**	Parvalbumin	-	10±1

Data are presented as mean value±S.D. *N*(peptide)=average number of peptides across all analysed samples, *N*(repetition)=9

**Table 4:** Selected proteins identified in raw and cooked salmon meat

**Access code**	**Protein**	***N*(peptide)**	
raw	cooked
**A0A1S3QIW0**	Myosin heavy chain, fast skeletal muscle-like	156±16	156±13
**A0A1S3NZ45**	Titin-like	137±15	83±18
**A0A1S3NZK3**	Nebulin isoform X11	67±8	90±17
**B5DG55**	Alpha-1,4 glucan phosphorylase	60±5	35±4
**A0A1S3NEY1**	Calcium-transporting ATPase	38±4	36±5
**A0A1S3SB73**	Actin, alpha cardiac	36±6	34±7
**B5DGP2**	Creatine kinase	28±3	26±3
**A0A1S2WZE0**	Fructose-bisphosphate aldolase	28±5	32±5
**B5DGR3**	Glyceraldehyde-3-phosphate dehydrogenase	27±4	29±3
**Q91472**	Fast myotomal muscle tropomyosin	25±4	-
**B5DGU1**	Pyruvate kinase	25±2	26±4
**A0A1S3LCK1**	ATP-dependent 6-phosphofructokinase	24±3	19±3
**Q7ZZN0**	Myosin regulatory light chain 2	21±3	19±2
**A0A1S3P5Q0**	Triosephosphate isomerase	20±1	-
**A0A1S3QZX8**	Glycogen debrancher	18±5	-
**A0A1S2WZE3**	2-phospho-d-glycerate hydro-lyase	-	33±4
**A0A1S3NGD5**	Myosin-binding protein C, fast-type-like	-	29±5
**B5DG39**	l-lactate dehydrogenase	-	13±2

Data are presented as mean value±S.D. *N*(peptide)=average number of peptides across all analysed samples, *N*(repetition)=9

The proteins with the largest number of identified peptides include myofibrillar proteins, which are most abundant in fish muscles: myosin, actin, nebulin, titin and tropomyosin. All proteins from this group are involved in a process of muscle contraction, for which they either directly provide or fulfil a regulatory function.

A large number of proteins from the second most represented group of proteins in fish meat were detected, namely sarcoplasmic proteins. Mainly enzymes belonging to the group of these water-soluble proteins were identified, *i.e.* creatine kinase (EC 2.7.3.2), glycogen phosphorylase (EC 2.4.1.1), SERCA (sarco/endoplasmic reticulum Ca^2+^ ATPase; EC 7.2.2.10), fructose-1,6-bisphosphate-aldolase (EC 4.1.2.13), glyceraldehyde-3-phosphate dehydrogenase (EC 1.2.1.12), pyruvate kinase (EC 2.7.1.40), lactate dehydrogenase (EC 1.1.1.27) and phosphofructokinase (EC 2.7.1.11). Other sarcoplasmic proteins such as myoglobin, α- and β-subunits of haemoglobin and parvalbumin were found. Parvalbumin is an important sarcoplasmic protein used for the authentication of fish meat and it is the main allergen of fish meat.

The third group of very little represented stromal proteins was represented by two types of collagens, type I and VI. In carp and salmon, significantly more peptides deriving from collagen were found in cooked meat.

#### Searching for characteristic peptide sequences

Evaluation of the LC-ESI-Q-TOF data against the UniProt database also enables a closer look at the peptide fragments. The found specific sequences of peptide fragments were analysed (similarly to the search for MALDI-TOF *m/z* markers) by the program pgAdmin 4, which works with the PostgreSQL database system (see Searching for species-specific markers).

#### Distinguishing between carp and salmon

The raw carp and salmon samples were evaluated separately from the cooked ones using the identical procedure. Firstly, the software tool pgAdmin 4 was used to determine which peptides were found in all samples of a species (*i.e.* nine samples: three biological individuals of a species and three technical replicates prepared from each individual). Then, the characteristic sequences for the respective species were determined, which were found in all samples of one species and at the same time in none of the samples of the other species. These characteristic sequences were approx. 400 for carp and 550 for salmon in the raw meat and approx. 400 sequences for carp and 600 for salmon in the cooked meat. At this point it is important to emphasise that "characteristic" in this case means specific only to the other compared species, *i.e.* sequences of carp are characteristic compared to salmon and *vice versa*. This is a proposal of a new method that could be used to identify fish meat after obtaining the measured data from a sufficient number of fish species. The results obtained for carp and salmon show that this method of identification can work. The advantage over MALDI-TOF MS is that it is possible to determine the specific peptide sequences responsible for differentiating the fish species and to identify the protein of origin from which these peptides originate.

The most important proteins for differentiating the species can be determined based on the number of found characteristic sequences. Table 5 shows the five proteins with the largest number of characteristic sequences for raw and cooked salmon and carp meat.

**Table 5:** Proteins containing the most species-characteristic sequences in raw and cooked carp and salmon samples

**Fish**	**Access code**	**Protein**	***N*(characteristic sequence)**	
			raw	cooked
**Carp**	A0A8C1U661	Myosin heavy chain, fast skeletal muscle-like	62	50
**Carp**	A0A8C2BNT8	Myosin, heavy chain b	32	24
**Carp**	A0A8C1U0K1	Nebulin	26	33
**Carp**	A0A2U9IYA4	Fructose-bisphosphate aldolase	19	-
**Carp**	A0A8C1WNY8	Calcium-transporting ATPase	19	16
**Carp**	A0A8C1N3F8	Creatine kinase M-type	-	20
**Salmon**	A0A1S3QIW0	Myosin heavy chain, fast skeletal muscle-like	90	86
**Salmon**	A0A1S3NZ45	Titin-like	86	59
**Salmon**	B5DG55	Alpha-1,4 glucan phosphorylase	37	-
**Salmon**	B5DGP2	Creatine kinase	31	30
**Salmon**	A0A1S3NZK3	Nebulin isoform X11	30	44
**Salmon**	A0A1S2WZE3	2-phospho-d-glycerate hydro-lyase	-	20

In raw carp samples, significantly more characteristic sequences originated from glycogen phosphorylase (10) than in cooked ones (4). Some proteins did not provide any characteristic sequences in the cooked carp meat samples – for example, the ryanodine receptor (a receptor associated with calcium channels) or the enzyme malate dehydrogenase. Both proteins had two characteristic sequences in the raw carp samples. The raw salmon meat contained more characteristic sequences for the enzymes phosphoglucomutase (8 *versus* 3) and glycogen debranching enzyme (8 *versus* 2) than the cooked meat.

On the contrary, cooked carp and salmon samples contained significantly more characteristic sequences originating from two proteins: parvalbumin and collagen. These two proteins were of great importance in distinguishing the cooked species (altogether around 20 characteristic sequences), while only two characteristic sequences from parvalbumin and one from collagen were found in raw salmon and none from these two proteins in the raw carp. In carp, 11 characteristic sequences of glyceraldehyde-3-phosphate dehydrogenase can be used for distinguishing cooked meat, but not a single one for distinguishing raw meat. Similarly, lactate dehydrogenase contains four characteristic sequences for distinguishing cooked meat, but none for raw meat. However, unlike parvalbumin and collagen, the same situation was not observed for these two enzymes in salmon samples.

#### Distinguishing between carp and salmon including the type of meat processing

Finally, an attempt was made to analyse the characteristic sequences including the type of processing (raw carp and salmon were compared simultaneously with their cooked meat). Only a few characteristic sequences specific to each sample type (*e.g.* raw carp) were identified: 17 characteristic sequences for raw carp, 40 for cooked carp, 10 for raw salmon and only 5 for cooked salmon. These markers in the form of characteristic sequences provide discriminatory value as they meet strict criteria: a given characteristic sequence is present in all nine samples of one material (*e.g.* raw carp), but it is not found in a single sample of the other materials (cooked carp, raw and cooked salmon). The characteristic sequences for each fish can be found in Supplementary material (Tables S1–S4).

Seven of the 40 characteristic sequences for cooked carp originated from parvalbumin, which demonstrates the two reported properties of parvalbumins: its interspecies variability and its thermostability. Owing to the interspecies variability, it is possible to distinguish between carp and salmon, and thanks to the thermostability, it is possible to find parvalbumin fragments in cooked meat. Another seven of the 40 characteristic sequences for cooked carp originate from glyceraldehyde-3-phosphate dehydrogenase. Characteristic sequences originally belonging to collagen were found in both cooked carp and salmon samples.

### Comparison of markers found by both mass spectrometric methods

A possible similarity between markers found with MALDI-TOF and LC-ESI-Q-TOF mass spectrometry was investigated. The *m/z* markers obtained from MALDI-TOF MS (see Table 1 and Table 2) were compared to the list of the characteristic peptide sequences (their *m/z*) obtained by LC-ESI-Q-TOF.

Since MALDI produce ions of peptides and proteins with a uniform charge of +1 and the ions generated by electrospray ionization can have multiple charges, it was not possible to directly compare the *m/z* values obtained by these two methods. The molecular mass of the peptide fragments were compared after charge subtraction. For MALDI-TOF, these values were easily obtained by subtracting the mass of one proton (MH^+^=1 Da), for LC-ESI-Q-TOF the mass of the peptide fragment was obtained directly from the results exported by Mascot 2.4.1. The tolerance was set to ±0.3 Da. Seven and three characteristic MALDI-TOF MS markers were obtained for raw carp and salmon samples respectively (see Table 1). Of these, three values for carp and none for salmon were found in the LC-ESI-Q-TOF markers in the form of characteristic sequences within the required tolerance. An overview of these values can be found in Table 6.

**Table 6:** Comparison of similarity of found markers between both mass spectrometric methods for raw and cooked meat samples of carp

**Type of meat**	**Species**	***M*(fragment/Da)**		**Peptide**	**Protein**
		MALDI-TOF	LC-ESI-Q-TOF		
**Raw**	Carp	1092.7	1092.56	K.GFTLPTTNSR.G	Creatine kinase, muscle b
	Carp	1262.6	1262.64	K.VAFNQVADIMR.A	LanC synthetase component C-like
	Carp	1308.7	1308.68	R.IDFDAFLPMLK.S	Myosin light chain 3, skeletal muscle isoform
**Cooked**	Carp	1092.7	1092.56	K.GFTLPTTNSR.G	Creatine kinase, muscle b
	Carp	1136.5	1136.61	K.NALAHAVQSAR.H	Myosin heavy chain, fast skeletal muscle
	Carp	1179.5	1179.55	R.LQTENGEFSR.Q	Myosin heavy chain, fast skeletal muscle
	Carp	2184.0	2184.03	K.GILGYTEDQVVST DFNGDVR.S	Glyceraldehyde-3-phosphate dehydrogenase

In cooked samples of the same fish species, 10 markers for carp and two for salmon were obtained using the MALDI-TOF method (see Table 2). Within the same tolerance, four out of ten carp markers were also found in the LC-ESI-Q-TOF data. However, not a single match was found for salmon.

## CONCLUSIONS

Using MALDI-TOF MS, species-specific markers were identified for each species in the form of characteristic *m/z* values. Additionally, markers that differentiate between raw and cooked meat of the same species were determined. Six markers with a high discriminatory power were found for carp, mackerel and pike, which would determine the type of processing (raw or cooked) in addition to species identification.

Only carp and salmon samples (raw and cooked meat) were analysed with LC-ESI-Q-TOF. A large number of species-specific amino acid sequences were found: in raw samples about 400 for carp and 550 for salmon, in cooked samples about 400 sequences for carp and 600 for salmon. The most characteristic sequences came from myosin, actin, nebulin, titin and sarcoplasmic enzymes. Both raw and cooked carp and salmon samples were included in the search for characteristic sequences. In this case, only a few dozen of characteristic sequences were identified for the given fish species and the method of meat preparation (*e.g*. for raw carp meat).

Since one of the most common types of adulteration of fish meat in general is species substitution, when meat of a more expensive species is replaced by meat of a cheaper species, the results of this work can be applied in food analysis. The identified species-specific markers have potential use in the assessment of the authenticity and taxonomic origin of fish products to ensure adequate quality and safety, especially in cases where morphological characteristics are lost during the processing of fish meat, or DNA and protein are degraded due to high temperatures during cooking.

## Appendix

**Table S1:** Characteristic peptides for raw carp samples

**Protein origin**	**Peptide**	***m/z***
**Actinin alpha 3a; Actinin alpha 3b; Alpha-actinin-3**	R.FAIQDISVEETSAK.E	769.39
**A kinase (PRKA) anchor protein 12b**	K.EESQAESKAEPK.V	666.82
**Alpha-tropomyosin; Tropomyosin alpha-1 chain-like; Tropomyosin alpha-1 chain-like**	K.DAQEKLELAEK.K	637.33
**ATPase sarcoplasmic/endoplasmic reticulum Ca^2+^ transporting**	K.FTLEFSR.D	450.23
**ATP citrate lyase b**	K.LSTIEFK.S	419.24
**Phosphorylase, glycogen, muscle A**	R.HLEIIYEINRR.H	485.94
**EF-hand calcium binding domain 7**	R.DIFDFAALK.E	520.27
	K.AIGGIILTASHNPGGPSGDFGIK.F	727.05
**Glycogen debranching enzyme-like**	R.VLDWINPTGR.E	585.81
**Malate dehydrogenase 2, NAD (mitochondrial)**	R.FTFSLLDAMNGK.E	672.33
**Myosin heavy chain, fast skeletal muscle-like; Myosin, heavy chain b**	K.SRVTFQLSAER.S	647.34
**SH3 domain binding glutamate-rich protein**	K.QQDVVGFLEALK.I	673.87
**Uncharacterized protein**	K.TLITDTVFK.I	519.30
**Uncharacterized protein**	-.MLMSHLEEPK.L	623.79
**Uncharacterized protein**	R.LNVSSTVTSTVLK.I	674.89
**Uncharacterized protein**	R.YFLTLENVTGSK.T	686.36
**Uncharacterized protein**	R.YSVTGLETGAEYK.F	709.34

**Table S2:** Characteristic peptides for cooked carp samples

**Protein origin**	**Peptide**	***m/z***
**Adenosine monophosphate deaminase 1 (isoform M)**	K.LAGWFNK.H	418.23
**Adenylate kinase isoenzyme 1**	K.IGAPALLLYIDAKAETMVQR.L	725.07
**Calsequestrin 1a**	K.SQKSEHYQEYEDAAEEFHPHIK.F	676.31
**Glucose-6-phosphate isomerase b**	K.ILVANFLAQTEALMK.G	831.46
	K.SITDVVNVGIGGSDLGPLMVTEALKPYSK.G	987.52
**Glyceraldehyde-3-phosphate dehydrogenase**	K.VIHDNFVIIEGLMSTVHAITATQK.T	660.10
	R.VCDLMAHMASKE.-	667.80
	R.VIISAPSADAPMFVMGVNHEKYDNSLK.V	734.12
	R.VIISAPSADAPMFVMGVNHEK.Y	738.37
	K.WGDAGANYVVESTGVFTTIEK.A	748.70
	R.SSIFDAGAGIALNDHFVK.L	931.47
	K.AAADGPMKGILGYTEDQVVSTDFNGDVR.S	976.13
**Collagen, type I, alpha 2**	R.GNPGPAGALGAQGPIGNR.G	802.42
	R.GPIGNIGMPGMTGPQGEAGR.E	956.95
	R.GPLGNIGMPGMTGPQGEAGR.E	956.95
**Creatine kinase, mitochondrial 2a (sarcomeric)**	K.TVGMVAGDEESYEVFAEIFDPVIKDR.H	972.80
**Creatine kinase, muscle b**	K.DIYNKLR.S	461.26
	K.GFTLPTTNSRGER.R	479.25
	K.VLTKDIYNK.L	547.32
**Enolase 3, (beta, muscle)**	K.FTGSVDIQVVGDDLTVTNPK.R	1053.04
**Myomesin 1a (skelemin)**	K.SDDVLIFDIGK.I	611.32
**Myomesin 1b**	K.ATNQSSLVLIGDVFK.Q	796.43
**Myomesin 2a**	R.FVVHGLVPGDTYVFR.V	569.30
**Myosin binding protein Hb**	K.VNLVVPFSGKPQPVVSWTK.D	694.73
**Myosin heavy chain, fast skeletal muscle-like**	R.TLEDQLSEIKTK.S	702.88
	R.ARLQTENGEFSR.Q	704.35
**Nebulin**	R.SDAVYKADLEWIR.G	522.60
	R.DIASDYKYK.L	551.77
**Parvalbumin-2-like**	K.SGFIEEDELKLFLQNFAAGAR.A	785.73
**Parvalbumin-7-like;** **Parvalbumin 7**	K.FFDVVGLK.A	462.76
	K.IGIDEFEALVHE.-	686.34
**Parvalbumin beta-like; Parvalbumin**	K.AFAIIDQDK.S	510.77
	K.IGVDEFTALVK.A	596.33
	K.AFAIIDQDKSGFIEEDELK.L	723.36
	K.AFAIIDQDNSGFIEEEELKLFLQNFK.A	765.39
**Pyruvate kinase M1/2b**	K.TTGSAFIQTQQMHAAMAETLLEHLCLLDIDSEPTVSR.N	1015.24
**Rhotekin 2a**	R.NIATRSTVSSCSSLAMEIK.R	500.26
**Triosephosphate isomerase 1b**	K.FFVGGNWK.L	477.74
**Troponin C type 2 (fast)**	K.AAFDMFDTDGGGDISTKELGTVMR.M	845.38
**Uncharacterized protein**	K.AGTKIELPADITGKPEPK.V	467.01

**Table S3:** Characteristic peptides for raw salmon samples

**Protein origin**	**Peptide**	***m/z***
**Alpha-1,4 glucan phosphorylase**	R.HLEIIYEINRR.F	485.94
**ATP-dependent 6-phosphofructokinase**	R.TFILEVMGR.H	533.29
**Glycerol-3-phosphate dehydrogenase**	K.NIVAVGAGFCDGLGFGDNTK.A	977.97
**Malate dehydrogenase**	R.FTFSVLDAMNGK.E	665.33
**Myosin-7-like**	M.EGDLNEMEIQLSHSNR.Q	624.62
	-.MEGDLNEMEIQLSHSNR.Q	673.63
**Myosin-binding protein C, fast-type-like**	K.LLDDYHVVVGER.V	472.25
**Titin-like**	R.FSLTIFR.A	442.25
	R.VLDSPSMPANFAIK.E	745.38
**TSC22 domain family protein 3**	-.MSTEIFK.T	428.22

**Table S4:** Characteristic peptides for cooked salmon samples

**Protein origin**	**Peptide**	***m/z***
**Annexin**	R.SLLLALVQAK.R	528.34
**Collagen alpha-3(VI) chain-like isoform X1; Collagen alpha-3(VI) chain-like isoform X2; Collagen alpha-3(VI) chain-like;** **Collagen alpha-3(VI) chain-like**	R.SQEGVPQMLILLSGGR.S	562.30
**Protein S100**	K.DLLNAELGEIMGK.N	701.86
**Troponin C, skeletal muscle**	K.NADGMLDFDEFLK.M	757.84
**Voltage-dependent anion-selective channel protein 3; Voltage-dependent anion-selective channel protein 3**	K.LTLSALIDGK.N	515.81
